# The Anti-tumoral Properties of Orexin/Hypocretin Hypothalamic Neuropeptides: An Unexpected Therapeutic Role

**DOI:** 10.3389/fendo.2018.00573

**Published:** 2018-09-27

**Authors:** Alain Couvineau, Stéphanie Dayot, Pascal Nicole, Valérie Gratio, Vinciane Rebours, Anne Couvelard, Thierry Voisin

**Affiliations:** INSERM UMR1149/Inflammation Research Center (CRI), Team “From Inflammation to Cancer in Digestive Diseases” Labeled by “La Ligue Nationale Contre Le Cancer,” Paris-Diderot University, DHU UNITY, Paris, France

**Keywords:** orexins, neuropeptides, GPCR, cancer, inflammation, gastroenterology, signaling pathway

## Abstract

Orexins (OxA and OxB) also termed hypocretins are hypothalamic neuropeptides involved in central nervous system (CNS) to control the sleep/wake process which is mediated by two G protein-coupled receptor subtypes, OX1R, and OX2R. Beside these central effects, orexins also play a role in various peripheral organs such as the intestine, pancreas, adrenal glands, kidney, adipose tissue and reproductive tract.In the past few years, an unexpected anti-tumoral role of orexins mediated by a new signaling pathway involving the presence of two immunoreceptor tyrosine-based inhibitory motifs (ITIM) in both orexin receptors subtypes, the recruitment of the phosphotyrosine phosphatase SHP2 and the induction of mitochondrial apoptosis has been elucidated. In the present review, we will discuss the anti-tumoral effect of orexin/OXR system in colon, pancreas, prostate and other cancers, and its interest as a possible therapeutic target.

## Introduction

Since its discovery in 1998 (1, 2), the role of orexins also named hypocretins has been extensively studied in the central nervous system (CNS) (3). Literature analysis revealed that about 2,000 articles have been published (Pubmed source, 1978 articles on July, 2018) demonstrating the great interest of “orexin” field in its role in the central nervous system. In contrast, the study of orexins in peripheral systems has been much less investigated, with only a hundred of articles published (Pubmed source, 103 articles on July, 2018). This great interest in central action of orexins was directly associated to their discovery in hypothalamus (1, 2). Orexins (OxA/hypocretin-1 and OxB/hypocretin-2) are two neuropeptides isoforms produced by the same prepro-orexin precursor (2). These two peptides have been shown to be involved in multiple CNS processes, including energy homeostasis, reward seeking, and drug addiction and the regulation of the sleep/wakefulness state which represents the major central effect of orexins, (4, 5). In human, narcolepsy type 1 (also known as narcolepsy with cataplexy) is the main pathology associated to a misregulation of orexins production caused by the loss of orexin neurons and characterized by a decreased ability to regulate sleep/wake cycles (6, 7). As mentioned above, orexins also play a role in various peripheral organs such as the intestine, pancreas, kidney, reproductive tract, adipose tissue and adrenal glands (8), although their roles remain controversial (9). Expression of orexins in peripheral tissues has been investigated using immunohistochemistry and/or RT-PCR techniques. Orexin have been detected in gastrointestinal tract, including colon (10), and pancreas (11), adrenal glands (12), kidney (11), adipose tissues (12) and reproductive tract including testis (13), and prostate (14). It should be noted that the analysis of literature related to orexins expression in peripheral tissues, revealed a large variability in term of orexins level and/or none expression. This could be related to the used tools, in particular the specificity of antibodies and/or RT-PCR, which reflected only the presence of preproorexin transcripts. The determination of basal circulating orexins concentration indicated a range of 2–45 pM (15, 16) which was about 1,000 times less in term of concentration than the IC_50_ of orexin receptors estimated to few 10 nM (17).

**Figure 1:**
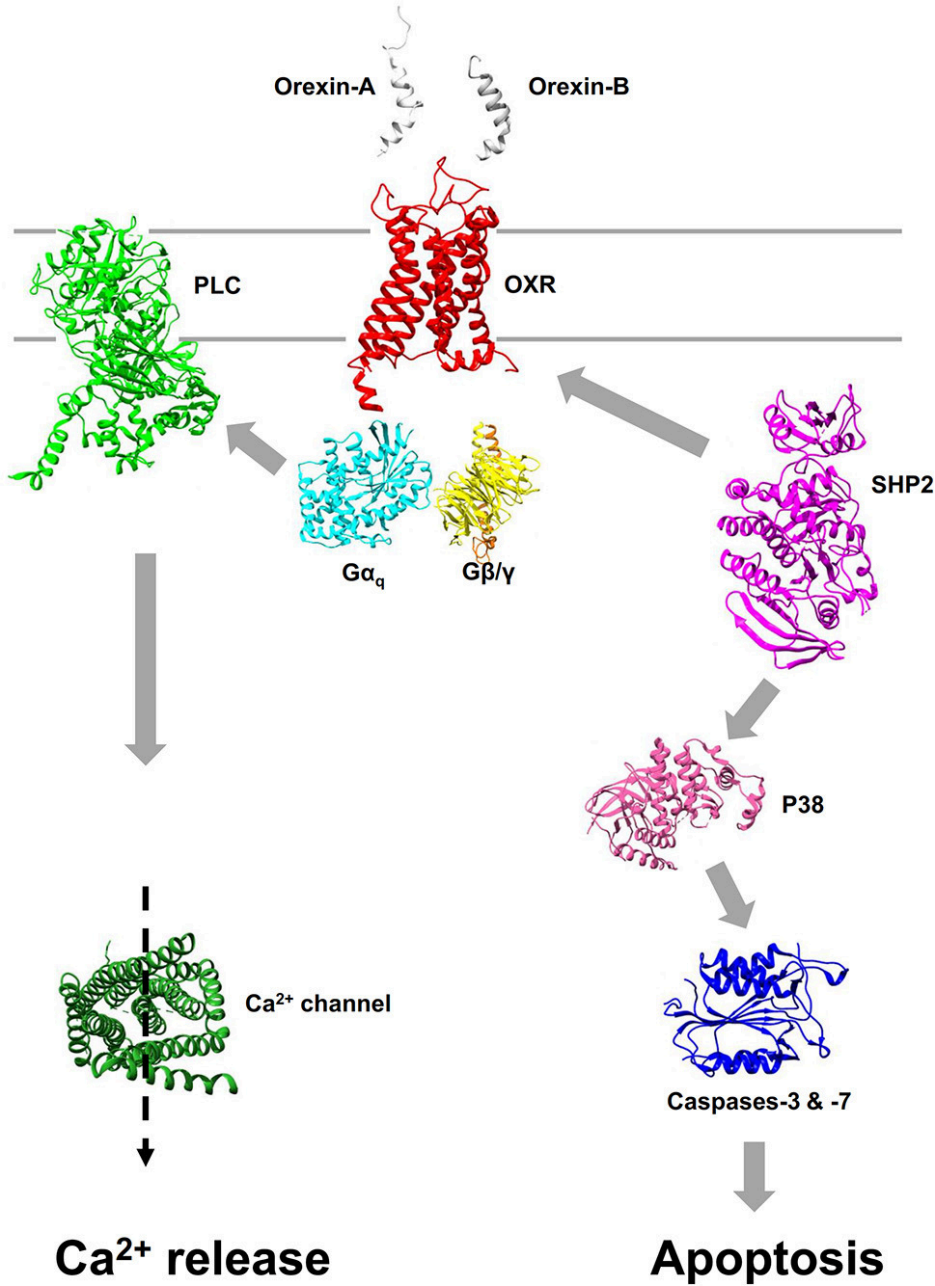
Pro-apoptotic signaling pathway induced by orexins. Interaction of orexins with OX1R or OX2R leads to promote the dissociation of heterotrimer G_q_ into α_q_ and β/γ subunits. The canonical Ca^2+^ signaling pathway stimulated by orexins results of phospholipase C (PLC) activation mediated by α_q_ subunit **(Left)**. Apoptosis induced by orexins is mediated, independently of canonical Ca^2+^ signaling pathway, by the recruitment of the phosphotyrosine phosphatase SHP2 leading to the activation of p38 cascade and activation of caspases-3 and -7 *via* the mitochondria apoptosis pathway **(Right)**. All structures were obtained in Protein Data Bank (PDB).

Orexins mediate their biological effects by interaction with two G-protein coupled receptors (GPCRs) subtypes, OX1R, and OX2R (also named Hcrtr-1 and Hcrtr-2, respectively) (18, 19), leading to the intracellular calcium releasing involving the Gq pathway (Figure 1). Although stimulation of orexin receptors predominantly leads to an increase in intracellular free calcium ions level, other signaling second messengers/pathways, i.e., cAMP, MAPK-Erk1/2, PI3K-Akt and JNK are also involved in orexins actions (19).

GPCRs characterized by seven α-helices transmembrane domains, belongs to the largest family of cell surface receptors with over 800 members in the human genome which are involved in the mainly pathophysiological actions (20). Classically, it was admitted that their major physiological actions were mediated “exclusively” by the G-protein signaling pathway, including effector stimulation and/or inhibition, desensitization and cellular internalization (21, 22). However, since several years, it has seen an increasing trend that many GPCRs action can also be mediated by other transduction mechanisms leading to a rich set of new physiopathological functions (20). Among their new roles, GPCRs are often overexpressed/underexpressed in tumor cells and also involved in the progression and/or initiation of cancer by inhibiting or stimulating proliferation and/or apoptosis (23, 24). In this review, we focus on the expression and anti-tumoral properties of OX1R in different cancers as gastrointestinal cancers (colon and pancreatic cancers) and prostate cancer, including their potential roles as therapeutic targets.

## Colon cancers

Colorectal cancer is the third most common cancer in men and the second most common in women, represents almost 10% of the annual global cancer incidence (25). Incidence rates of colorectal cancer show a strong positive gradient with an increasing level of economic development. Approximately 60% of patients with colorectal will present liver metastases during the course of disease (26). The only option to fight against the appearance of hepatic metastases of the colorectal cancers is the surgical resection. However, the rate of second recurrence stays of 75 % after metastasectomy (27). The patient's survival is dependent on the stage at diagnosis. It is positive for the premature lesions (Stage I), intermediate for stages II and III and poor for the metastatic stages. A post-operative chemotherapy is proposed for stages II and III. In the case of the rectal cancer, the association of a chemotherapy and a radiotherapy strongly reduced the relapse incidences and prolonged patients' survival (27). Since the 1980s, the global survival of the metastatic diagnosed patients increased by the use of new cytotoxic molecules (i.e., oxaliplatin, irinotecan), combined with anti-angiogenic and anti-EGFR molecules (28). To date, this survival was strongly increased by the combination of the three most effective chemotherapeutic agents (fluorouracil, irinotecan and oxaliplatin) (29).

Tumor-suppressor genes and oncogenes was identified as key genes whose mutations or altered expression are associated with colorectal cancer (30). Colon cancer initiation and progression, which are under these genes control, are also regulated by growth factors or hormones present in the tumor environment which action are mediated trough tyrosine kinase receptors or G protein-coupled receptors (GPCRs) (31). Many GPCRs were similarly expressed in normal colon epithelial cells, others are overexpressed and some of them are ectopically expressed in cancer cells (31–36). The peptide hormones mediated-growth effects such as gastrin (34) or neurotensin (33), serine proteases such as thrombin (35) or trypsin (37) or lipids such as lysophosphatidic acid (38) or prostaglandin E2 (39) are promoted through GPCRs. Activation of these GPCRs activation leads tumoral growth via G protein transduction pathways and/or by transactivating the tyrosine kinase epidermal growth factor receptor (EGFR) (40, 41). The environment of primary colon tumors is rich in growth factors, however the existence of growth inhibitory factors for colon cancer is not well documented. In order to determine these inhibitory factors, the screening of the ability of different peptide hormones and neuropeptides to inhibit colon cancer growth was investigated (42). Twenty-six peptides were tested, including orexins which were present in few peripheral tissues including the gastrointestinal tract (10). The screening, using the human colon cancer cell line HT-29 grown in standard trophic conditions shows that only the two related peptides OxA and OxB was able to inhibit tumoral cell growth (42). Orexins do not modify cell cycle and proliferation, but activate cell death by apoptosis with a plasma membrane phosphatidylserine externalization, chromatin condensation and DNA fragmentation (42–44). Only OX1R, and not OX2R, is expressed in HT-29 cells and is involved in the orexin-induced apoptosis. Orexins promote cell death described by a mitochondrial cytochrome c release and caspase-3 and caspase-7 protease activations (42, 44). The ability of orexins to activate a robust apoptosis has been shown in 9/10 (90%) different human colon cancer cell lines (44). Conversely, orexins do not trigger apoptosis in explant cultures of human normal colonic mucosa demonstrating that the orexin-induced apoptotis appeared during the colonic epithelial cell oncogenesis (44). However, preproorexin and OxA has been detected in normal total colon (10, 11). In contrast, no detection of preproorexin was observed in normal and tumoral colonic epithelia (44). Moreover, in preclinical models, the tumoral development of xenografted tumor from HT-29 cells which expressed OX1R or HCT-116 cells which do not expressed OX1R were identical (44). These observations indicate that endogenous OxA, present in colon but not in colonic epithelium, have no impact on tumoral development.

The drug resistance occurrence is a primary cause of chemotherapy failure. The 5-fluorouracil (5-FU) represents the “gold standard” molecule used in treatment of colon cancer. The OX1R expression was investigated in the HT-29-FU colon cancer cell line model, developed after a long-term 5-FU exposure clonal cells resistant against the drug (45). The OX1R expression, orexins-induced apoptosis and subsequent growth inhibition were similar in resistant HT-29-FU cells and sensitive initial HT-29 cells (44), suggesting that orexins-induced apoptosis persists in resistant cells (44). Moreover, OX1R is expressed in 100% of primary colorectal tumors resected from patients (38 different colorectal cancers) whatever their stages and in 10 hepatic metastases and in human colon cancer cell lines established from lymph nodes, ascite, and lung metastases tested (44).

**Figure 2:**
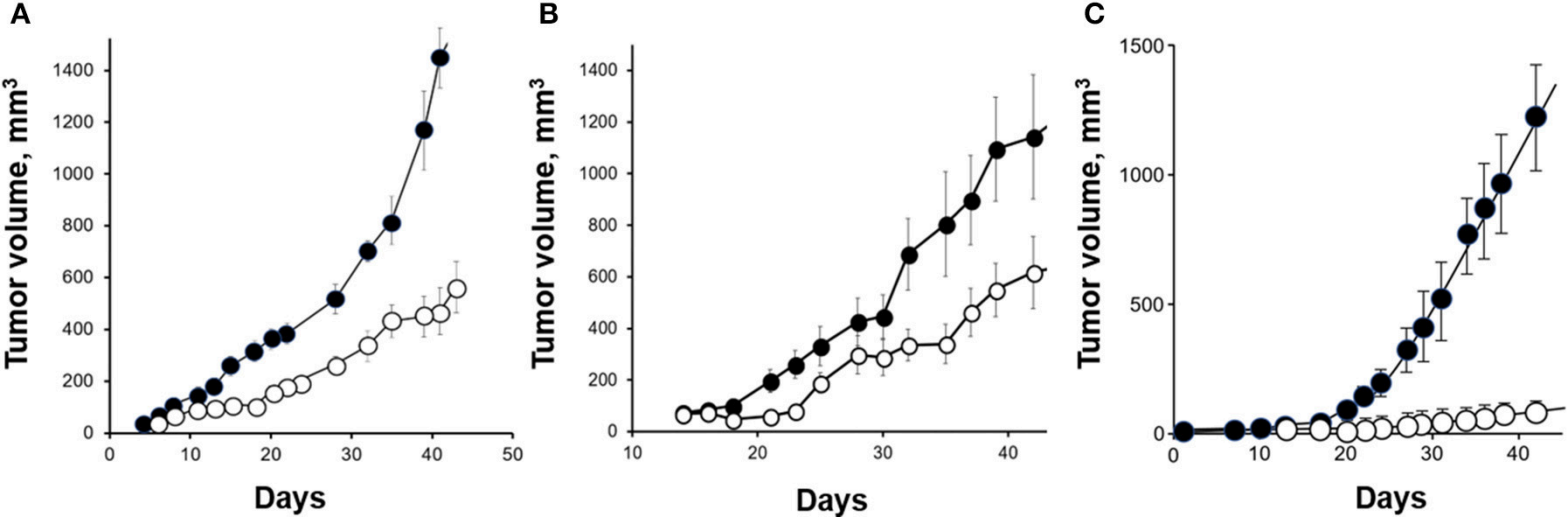
Anti-tumoral effect of OxA in preclinical mouse models. **(A)** Nude mice were xenografted with the colon adenocarcinoma cell line, HT-29 and then, treated by 1 μmoles of OxA/Kg intraperitoneally (ip) injected. The tumor development was determined by measurement. **(B)** Nude mice were xenografted with the pancreatic adenocarcinoma cell line, AsPC-1, and ip injected with 1 μmoles of OxA/Kg. **(C)** Nude mice were xenografted with the prostate cancer cell line, DU145 and treated with 1 μmoles of OxA/Kg. (•), control mice injected with PBS; (◦), treated mice injected with OxA. The sources of graphs were based on Voisin et al. (44), Dayot et al. (46), and Chartrel et al. (47).

The efficiency of *in vivo* orexin treatment was addressed using human colon cancer cells xenografted in nude mice. When human colon cancer cells were xenografted in nude mice, daily OxA administration strongly slowed the tumor growth and even reversed the development of established tumors when administered 7 days after cell inoculation. After a 15-days orexins treatment, the tumor volume is decreased by 80% (Figure 2) (44). It was shown that orexins treatment reduces tumor growth *in vivo* by promoting apoptosis, through activation of caspase-3 (44).

The OX1R-driven apoptosis even though calcium pathway could not be explained only by the classical Gq-mediated calcium response. Two tyrosine-based motifs (ITIM) were identified in OX1R which have a crucial role in OX1R-driven apoptosis (Figure 1) (43, 48). The ITIM is not considered to be a GPCRs' signature, but represents a hallmark of immune inhibitory receptors (49). After activation of OX1R by orexins, the two ITIMs are phosphorylated on tyrosine residue (43, 48). It should be noted that the classical Gq-mediated activation of phospholipase C is not involved in this process. When orexins promoted tyrosine phosphorylation of ITIMs, OX1R recruits and activates the phosphotyrosine phosphatase SHP2 which is crucial in the orexin-induced apoptosis process (43, 48). The intracellular signaling pathway downstream of SHP2 includes the p38 mitogen-/stress activated protein kinase phosphorylation, which leads to the proapoptotic protein Bax translocation in the mitochondria, the apoptosome formation, caspase-3 and caspase-7 activation and cell death (Figure 1).

## Pancreas cancer

Pancreatic ductal adenocarcinoma (PDAC) is the tenth most common cancer sites in terms of frequency and is the fifth cause of cancer mortality (50, 51). Moreover, the projection cancer incidence and deaths in 2030 indicate that this cancer could become the second cause of cancer-related death (52). Invasive PDAC which carries a very poor prognosis (5-year survival rate < 8%), is rarely surgically resectable and <20% of patients undergoing a curative surgery. In addition, PDAC is one of the most chemotherapeutic drug-resistant tumors (53). The high therapeutic resistance of PDAC can be explained by immunodepression, hypoxic microenvironment and a pronounced fibrotic reaction consisting of proliferating stromal cells together with collagen-rich extracellular matrix (53). This fibrotic stroma can account for more than 80% of the tumor mass (54), has been shown to limit the delivery of therapeutics, and contribute to tumor progression and drug resistance (55). Despite increased knowledge in the etiology of PDAC, successful therapeutic strategies are still very poor.

**Figure 3:**
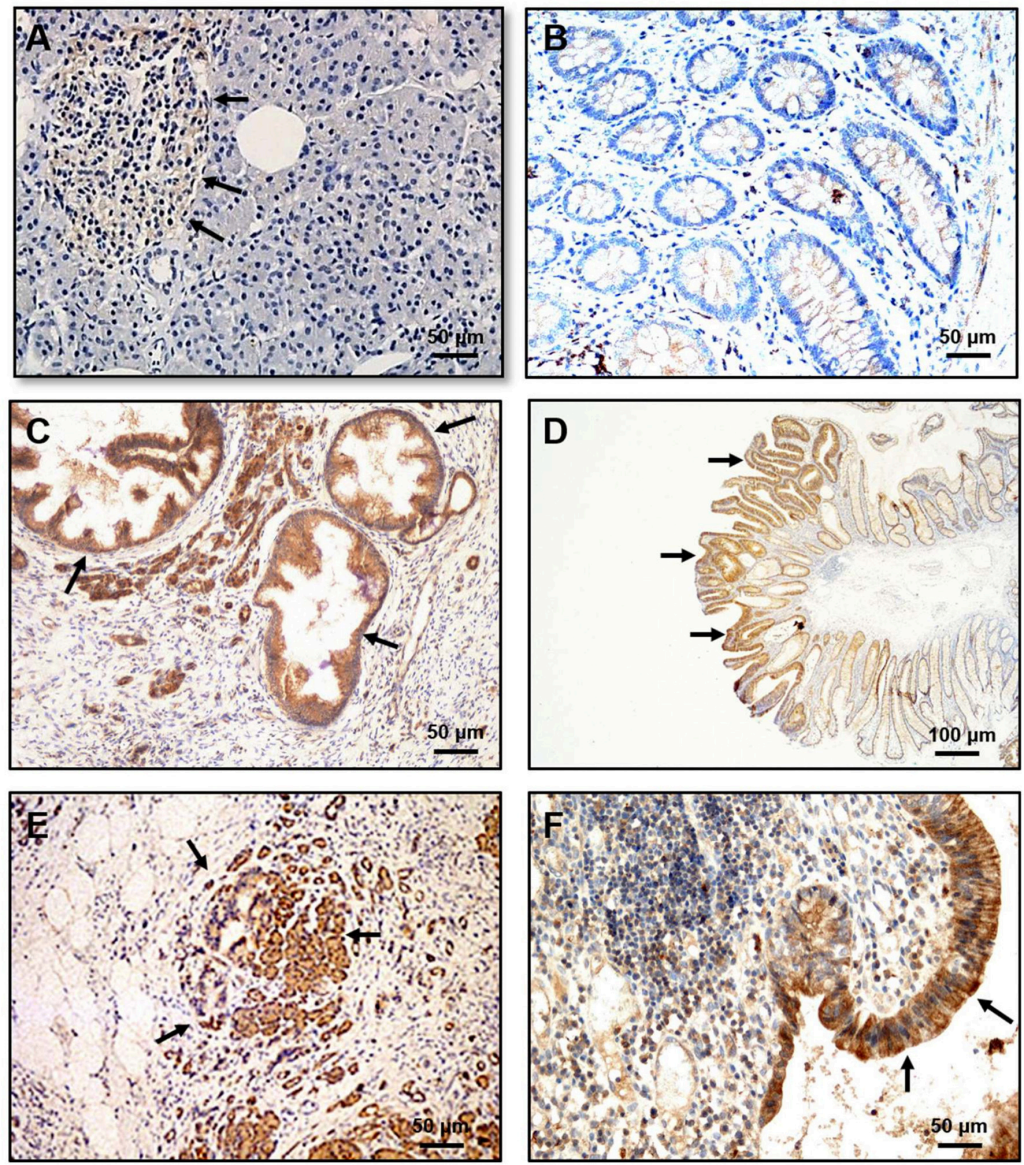
Immunohistochemical expression of OX1R in PanIN, dysplastic colonic polyp, pancreatitis and ulcerative colitis (UC). **(A)** OX1R was not detected in the normal pancreas either in normal duct and acinar cells. **(B)** OX1R was not detected in colonic mucosa. **(C)** OX1R was expressed in PanIN lesions. **(D)** OX1R was expressed in dysplastic cells present in colonic polyps. **(E)** OX1R immunostaining of colonic mucosa from patients with pancreatitis. **(F)** OX1R immunostaining of colonic mucosa from patients with UC. Arrows indicated the OX1R expression. Bar = 50 μm for **(A, B C, E, F)**; Bar = 100 μm for **(D)**. The sources of graphs were based on Voisin et al. (44), Dayot et al. (46), and Messal et al. (56).

Recently, the OX1R expression was detected in 70/73 human PDAC (96 %) and in 83/103 human pancreatic neuroendocrine tumors (46). It should be noted that OX1R was not expressed in normal pancreas (acini and ducts) except in the Langerhans islets (Figure 3A) in which orexins could play a role in insulin secretion (8). This expression in tumoral tissue was independent of patient age, gender, tumor size, and lymph node metastasis (46). The use of AsPC-1 cell line derived from human PDAC revealed that OxA was able to strongly inhibit cell growth by the SHP2-induced apoptosis (46). Moreover, the treatment by OxA of tumor slices obtained from patients and maintained in culture, induced the activation of caspases-3 in tumoral tissue demonstrating that OxA was able to induce apoptosis in PDAC (46). In preclinical model consisting in sub-cutaneous xenografted AsPC-1 cells in nude mice, OxA reduced significantly the tumor growth (Figure 2B). This tumor regression was also observed in tumors established 14 days prior OxA treatment (46). For translational studies, the patient-derived xenograft (PDX) model was frequently used. In such models, the tumoral fragments, or the isolated cells from the patient's cancer were implanted in immunodeficient mice, OxA was also able to drastically reduce the tumor growth derived from PDAC indicating its potential therapeutic interest (46). Previously report revealed the expression of OxA in endocrine pancreas (11). However, the presence of endogenous OxA does not seem to be involved in anti-tumoral effect of exogenous OxA since the tumoral development of xenografted tumor from AsPC-1 cells which expressed OX1R or HPAF-II cells which do not expressed OX1R were very similar (46). Moreover, the concentration of circulating orexins was very low (about 40 pM) to functionally activate OX1R.

As mentioned in the introduction, orexins and their receptors have been extensively studied in CNS notably in sleep regulation. In this context, many academic and pharmaceutical laboratories have focused their researches in the development of molecules able to improve sleeping regulation, in particular, in insomnia (57, 58). A lot of antagonists were developed and sub-divided into two classes named single orexin-receptor antagonists (SORAs) and dual orexin-receptor antagonists (DORAs). Among them, the SORA small molecule SB-408124 or SB-334867 was shown to be specific of OX1R (59, 60) and JNJ-42847922 specific of OX2R (61). However, the main molecule development, in particular antagonists, was related to the sleep-wakefulness actions of orexins leading to the design of DORA such as SB-649868, almorexant (ACT-078573) (62) and suvorexant (MK-4305) for which the U.S. Food and Drug Administration (FDA) approved the use for the treatment of insomnia (63). In parallel, the development of OXR agonists was substantially much lower. Despite some attempts, no OX1R agonist was actually available (64) and only few OX2R agonists have been developed such as the non-peptidic molecules YNT-185 (65), OX2R-agonist 26 (66), and the peptide agonist SB-668875 (67) but these molecules are poorly documented.

Surprisingly, the use of suvorexant or almorexant on PDAC cell line, AsPC-1 revealed that these molecules inhibited the cellular growth by apoptosis induction (46). Almorexant appearing to be more potent than suvorexant to induce this inhibitory effect. Moreover, intraperitoneal injections of almorexant in xenografted mouse model, induced a significant reduction in tumor size (>50%) similar to the anti-tumoral effect of OxA in the same conditions (46). As shown in Figure 1, OxA and OxB activated two signaling pathways, including: (1) the canonical intracellular Ca^2+^ release effect mediated by the Gq protein which was totally inhibited in the presence of SORA and/or DORA (46) and; (2) the recruitment of SHP2 mediated by the phosphorylation of ITIM sites leading to the intrinsic apoptosis mediated by the p38 signaling pathway which was not affected by DORA (46). Recently, structure-function relationship analysis of OxB evidenced that some residues of the peptide discriminated between proapoptotic and calcium pathways (17). Likewise, almorexant which binds to OX1R with the similar affinity than OxA could discriminate these two signaling pathways demonstrating the existence of two independent molecular activation of the OX1R. These observations, suggest that almorexant (and also suvorexant) belong to ligand-biased family (68). Therefore, OX1R antagonists, which was prescribed for insomnia could be used in the anti-tumoral therapy as full agonist.

## Prostate cancer

With about 71,000 new cases of prostate cancer in France each year, this cancer represents the most commonly diagnosed malignant tumor for men in the Western world, far ahead lung cancers and colorectal cancers (69). Despite the progress in screening, prostate cancer is the second cause of cancer-related mortality (70) and is associated with resistance to chemo-hormonal therapy in the metastatic setting. It should be noted that, more one in nine men will disclose a prostate cancer during his life. Because androgens stimulate the tumor growth, androgen ablation therapy represents the first line of treatment of advanced cancer inducing an effective tumoral regression (71). However, over the years an androgen resistance named castration-resistant prostate cancer (CRPC) develops. The cause of this resistance remains still unclear, but some investigations revealed an overexpression/amplification of androgen receptors (AR), a gain-of-function of AR, a production of AR variants having constitutive properties, an overexpression of co-factors of AR and an intra-tumoral production of androgen (72). In addition to the major role of the androgen/AR system in prostate cancer, various GPCRs are involved in the development and progression of prostate cancer (73). These GPCRs include gonadotropin hormone receptors [luteinizing hormone receptor (LHR) and follicle-stimulating hormone receptor (FSHR), peptide receptors neurotensin receptor (NTR), bombesin receptor (BBR), endothelin-1 receptor (ETR), oxytocin receptor (OXTR), and ghrelin receptor (GHSR)], protease receptors [thrombin receptors (PARs)] and neuropeptide receptors [neuropeptide Y receptor (NPYR)], vasoactive intestinal peptide receptor (VPAC), and pituitary adenylyl cyclase activating peptide (PAC1). This partial list of GPCRs and their ligands promoted proliferation, migration, invasion, mitogenic signaling, and neuroendocrine differentiation of prostate cancer cells (73–81). Moreover, GPCRs expressed and/or overexpressed in prostate cancer are able to engage a cooperative crosstalk with growth factor receptors such as epidermal growth factor receptor (EGFR) (82). This transactivation mediated by GPCRs such as PAR receptors leads to the cleavage of EGF-like transmembrane ligands [EGF, transforming growth factor α (TGFα) …] by cancer cells. Thereby, a soluble biologically active growth factor was produced and induced mitogenic effects mediated by EGFR (40). In contrast, few GPCRs were involved in the inhibition of growth and/or in apoptosis of prostate cancer cells. The gonadotropin-releasing hormone receptor (GnRH) was expressed in human malignant prostate tumors where its activation induced an anti-tumoral activity mediated by p38 MAPK and protein tyrosine phosphatase (73). However, nothing is known about neuropeptides and their receptors in anti-tumoral properties in prostate cancer. OX1R but not OX2R was highly expressed in high grade advanced prostate cancer (CaP) whereas this expression was much lower in low grade cancer (83). Inversely, in benign prostatic hyperplasia (BPH), OX1R expression was mostly absent and mainly confined in scattered cells (83). It should be noted that OX2R seemed to be expressed in BPH, which was associated with a decrease of OxA serum concentration (84). The expression of OxA and its precursor was found in “fiber-like” stroma of prostate cancer tissues which did not correspond to nerve and smooth muscle fibers (83). In normal tissue, OxA was expressed in follicular exocrine epithelium (14) and also in hyperplastic epithelium. However, large areas of prostate epithelium were not immunoreactive (14). Moreover, the presence of OxA was never detected in cancerous foci whatever the cancer grade (83). Taken together these observations suggested that OX1R which is expressed in cancer cells was probably not activated by endogenous OxA produced by the prostate stroma and/or delivered by the blood circulation (83). OX1R was expressed in androgen-unresponsive cell line, DU145 in which OxA or OxB induced a significant apoptosis (83). In addition, OX1R was also expressed in androgen-responsive cell line LNCaP in which OxA induced an up-regulation of OX1R gene expression and inhibited cell survival (85). *In vivo* studies using xenografted mouse model with DU145 cells revealed that daily intraperitoneally injection of OxA induced a strong reduction of tumor volume (Figure 2C).

## Other cancers

Like the peripheral biological role of orexins that remains still under discussion (86), OXR expression and orexins actions in cancer have been poorly documented (87). Several lines of evidence indicated that OX1R/OX2R were expressed in various cancer cells, but their actions depended on the cancer types. It should be noted that the OXR expression was mainly determined using a great variety of antibodies (produced by various manufacturers) some of them have been identified as non-specific, in particular for anti-OX2R antibody which also recognized OX1R. In the same manner, the use of antibodies to detect the presence of OxA and/or OxB peptides in tissues was also questionable. Nevertheless, the possibility of OXR expression by other solid tumors is always under investigation. OX1R was expressed in neuroblastoma in which orexins treatment induced apoptosis (42). OX1R was also identified in cortical adenomas but its relation to apoptosis was not investigated (88, 89). In human hepatocellular carcinoma tissues (90), in gastric cancer cell lines, SGC-7901 and BGC-823, OxA seemed to enhance the proliferation and inhibited the apoptosis which is mediated by the ERK or AKT signaling pathway (91, 92), respectively. Moreover, OxA and cholecystokinin (CCK) inhibited the migration of colorectal cancer cell line, HT-29 mediated by heterodimerization of OX1R and CCK1R (93). In addition, OX2R was expressed in human pheochromocytomas and PC12 cells in which OxA and OxB stimulated (94) or inhibited (95) catecholamine secretion, in endometrial endometrioid carcinoma in which OxA and OxB had no effect on proliferation and/or apoptosis (96), and in human adrenocortical NCI H295R cells where OxA induced the phosphorylation of ERK1/2 and p38 (88). Taken together these observations indicated that OX1R and OX2R were expressed in various cancers. In contrast, in the corresponding healthy tissues such as colonic epithelium and pancreatic acini, OX1R was not expressed [Figure 3 and (49)]. It may be noted that OX1R is expressed in Langerhans's islets (Figure 3). In this context, an important question arises: is OX1R expressed at early stages of cancer development? As shown in Figure 3, the dysplastic cells present in colon polyps or pancreatic intraepithelial neoplasia (PanIN) lesions highly expressed OX1R indicating that the expression of the receptor occurred at a very early stage (46). Chronic inflammation, including intestinal bowel disease (IBD), pancreatitis, hepatic fibrosis… or metabolic syndrome which is close to chronic inflammation, represent a high-risk factor in the development of cancer (97). What is the role of OX1R expression in inflamed tissues? Various studies have demonstrated that orexins exercised neuroprotection effects and reduced cerebral neuroinflammation associated to post-stroke trauma (98, 99). Recently, Ogawa et al. demonstrated that OxA alleviated the survival of mice with endotoxin shock characterized by a systemic inflammation (100). Some reports revealed a relationship between orexinergic system and metabolic syndrome disorder (101). In ulcerative colitis (UC) and pancreatitis, OX1R was highly expressed in inflamed areas (Figure 3). Moreover, OxA was able to induce an anti-inflammatory effect in mice models reproducing UC or pancreatitis (56, 102). A recent study indicates a higher prevalence of immunopathological diseases, including purpura, multiple sclerosis, systemic lupus erythematosus, psoriasis, Crohn's disease, or ulcerative colitis, in narcoleptic patients (103). Besides, the anti-tumoral properties of OXR/orexins system, orexins could play an important role in chronic inflammation.

## Conclusion

These last two decades, orexins/OXR system has been extensively studied in CNS in particular in the regulation of sleep/wake. These intensive and fruitful investigations lead to development of therapeutic molecules which are prescribed to treat insomnia. Beside this innovative research in CNS, the orexins/OXR system has a potential benefit in peripheral physiopathology, especially in cancer, and chronic inflammatory diseases. These promising perspectives open up new fields of application in the development of new therapeutic agonist molecules (including peptides, small non-peptidic molecules, and functional agonist antibodies) and/or the use of molecules already developed such as almorexant, suvorexant…. In the future decade, the orexins/OXR system could constitute a crucial curative target in human cancers.

## Author contributions

SD, VG, PN, VR, and AnC have participated in the work and has proofread the manuscript. AlC and TV have written the manuscript. AlC was the head of the “from inflammation to cancer in digestive diseases” group.

### Conflict of interest statement

The authors declare that the research was conducted in the absence of any commercial or financial relationships that could be construed as a potential conflict of interest.

## References

[1] de Lecea L Kilduff TS Peyron C Gao X Foye PE Danielson PE. The hypocretins: hypothalamus-specific peptides with neuroexcitatory activity. Proc Natl Acad Sci USA. (1998) 95:322–27. 10.1073/pnas.95.1.322 9419374 PMC18213

[2] Sakurai T Amemiya A Ishii M Matsuzaki I Chemelli RM Tanaka H Orexins and orexin receptors: a family of hypothalamic neuropeptides and G protein-coupled receptors that regulate feeding behavior. Cell (1998) 92:573–85. 10.1016/S0092-8674(00)80949-6 9491897

[3] Laburthe M Voisin T. The orexin receptor OX(1)R in colon cancer: a promising therapeutic target and a new paradigm in G protein-coupled receptor signaling through ITIMs. Br J Pharmacol. (2012) 165:1678–87. 10.1111/j.1476-5381.2011.01510.x 21627633 PMC3372822

[4] de Lecea L Sutcliffe JG. The hypocretins/orexins: novel hypothalamic neuropeptides involved in different physiological systems. Cell Mol Life Sci. (1999) 56:473–80. 10.1007/s000180050446 11212299 PMC11146771

[5] Mieda M Yanagisawa M. Sleep, feeding, and neuropeptides: roles of orexins and orexin receptors. Curr Opin Neurobiol. (2002) 12:339–45. 10.1016/S0959-4388(02)00331-8 12049942

[6] Lin L Faraco J Li R Kadotani H Rogers W Lin X. The sleep disorder canine narcolepsy is caused by a mutation in the hypocretin (orexin) receptor 2 gene. Cell (1999) 98:365–76. 10.1016/S0092-8674(00)81965-0 10458611

[7] Chemelli RM Willie JT Sinton CM Elmquist JK Scammell T Lee C. Narcolepsy in orexin knockout mice: molecular genetics of sleep regulation. Cell (1999) 98:437–51. 10.1016/S0092-8674(00)81973-X 10481909

[8] Heinonen M V Purhonen A K Mäkelä K A Herzig K H. Functions of orexins in peripheral tissues. Acta Physiol. (2008) 192:471–85. 10.1111/j.1748-1716.2008.01836.x 18294339

[9] Voisin T Rouet-Benzineb P Reuter N Laburthe M. Orexins and their receptors: structural aspects and role in peripheral tissues. Cell Mol Life Sci. (2003) 60:72–87. 10.1007/s000180300005 12613659 PMC11138506

[10] Kirchgessner AL Liu M. Orexin synthesis and response in the gut. Neuron (1999) 24:941–51. 10.1016/S0896-6273(00)81041-7 10624957

[11] Nakabayashi M Suzuki T Takahashi K Totsune K Muramatsu Y Kaneko C. Orexin-A expression in human peripheral tissues. Mol Cell Endocrinol. (2003) 205:43–50. 10.1016/S0303-7207(03)00206-5 12890566

[12] Randeva HS Karteris E Grammatopoulos D Hillhouse EW. Expression of orexin-A and functional orexin type 2 receptors in the human adult adrenals: implications for adrenal function and energy homeostasis. J Clin Endocrinol Metab. (2001) 86:4808–13. 10.1210/jcem.86.10.7921 11600545

[13] Jöhren O Neidert SJ Kummer M Dendorfer A Dominiak P. Prepro-orexin and orexin receptor mRNAs are differentially expressed in peripheral tissues of male and female rats. Endocrinology (2001) 142:3324–31. 10.1210/endo.142.8.8299 11459774

[14] Valiante S Liguori G Tafuri S Campese R Monaco R Paino S. Expression of orexin A and its receptor 1 in the human prostate J Anat. (2013) 222:473–80. 10.1111/joa.12030 23425077 PMC3610039

[15] Arihara Z Takahashi K Murakami O Totsune K Sone M Satoh F. Immunoreactive orexin-A in human plasma. Peptides (2001) 22:139–42. 10.1016/S0196-9781(00)00369-7 11179609

[16] Sakurai S Nishijima T Takahashi S Yamauchi K Arihara Z Takahashi K. Clinical significance of daytime plasma orexin-A-like immunoreactivity concentrations in patients with obstructive sleep apnea hypopnea syndrome. Respiration (2004) 71:380–4. 10.1159/000079643 15316212

[17] Nicole P Couvineau P Jamin N Voisin T Couvineau A. Crucial role of the orexin-B C-terminus in the induction of OX1 receptor-mediated apoptosis: analysis by alanine scanning, molecular modelling and site-directed mutagenesis. Br J Pharmacol. (2015) 172:5211–23. 10.1111/bph.13287 26282891 PMC4687804

[18] Laburthe M Voisin T El Firar A. Orexins/hypocretins and orexin receptors in apoptosis: a mini-review. Acta Physiol. (2010) 198:393–402. 10.1111/j.1748-1716.2009.02035.x 19719798

[19] Leonard CS Kukkonen JP Orexin/hypocretin receptor signaling: a functional perspective. Br J Pharmacol. (2014) 171:294–13. 10.1111/bph.12296 23848055 PMC3904253

[20] Couvineau A Laburthe M. The family B1 GPCR: structural aspects and interaction with accessory proteins. Curr Drug Targets (2012) 13:103–115. 10.2174/138945012798868434 21777182

[21] Peterson YK Luttrell LM. The diverse roles of arrestin scaffolds in G protein-coupled receptor signaling. Pharmacol Rev. (2017) 69:256–97. 10.1124/pr.116.013367 28626043 PMC5482185

[22] Rajagopal S Shenoy SK. GPCR desensitization: acute and prolonged phases. Cell Signal. (2018) 41:9–16. 10.1016/j.cellsig.2017.01.024 28137506 PMC5533627

[23] Nieto Gutierrez A McDonald PH GPCRs: Emerging anti-cancer drug targets. Cell Signal. (2018) 41:65–74. 10.1016/j.cellsig.2017.09.005 28931490

[24] Moody TW Ramos-Alvarez I Jensen RT. Neuropeptide G protein-coupled receptors as oncotargets. Front Endocrinol. (2018) 9:345. 10.3389/fendo.2018.00345 30008698 PMC6033971

[25] Torre LA Bray F Siegel RL Ferlay J Lortet-Tieulent J Jemal A. Global cancer statistics 2012. CA Cancer J Clin. (2015) 65:87–108. 10.3322/caac.21262 25651787

[26] Valentini V van Stiphout RG Lammering G Gambacorta MA Barba MC Bebenek M. Nomograms for predicting local recurrence, distant metastases, and overall survival for patients with locally advanced rectal cancer on the basis of European randomized clinical trials. J Clin Oncol. (2011) 29:3163–72. 10.1200/JCO.2010.33.1595 21747092

[27] Siegel R Desantis C Jemal A. Colorectal cancer statistics 2014. CA Cancer J Clin. (2014) 64:104–17. 10.3322/caac.21220 24639052

[28] Van Cutsem E Tabernero J Lakomy R Prenen H Prausová J Macarulla T. Addition of aflibercept to fluorouracil, leucovorin, and irinotecan improves survival in a phase III randomized trial in patients with metastatic colorectal cancer previously treated with oxaliplatin-based regimen. J Clin Oncol. (2012) 30:2499–506. 10.1200/JCO.2012.42.8201 22949147

[29] Grothey A Sargent D Goldberg RM Schmoll H J. Survival of patients with advanced colorectal cancer improves with the availability of fluorouracil-leucovorin, irinotecan, and oxaliplatin in the course of treatment. J Clin Oncol. (2004) 22:1209–14. 10.1200/JCO.2004.11.037 15051767

[30] Markowitz SD Bertagnolli MM. Molecular origins of cancer: molecular basis of colorectal cancer. N Engl J Med. (2009) 361:2449–60. 10.1056/NEJMra0804588 20018966 PMC2843693

[31] Insel PA Sriram K Wiley SZ Wilderman A Katakia T McCann T. GPCRomics: GPCR expression in cancer cells and tumors identifies new, potential biomarkers and therapeutic targets. Front Pharmacol. (2018) 9:431. 10.3389/fphar.2018.00431 29872392 PMC5972277

[32] Laburthe M Rousset M Boissard C Chevalier G Zweibaum A Rosselin G. Vasoactive intestinal peptide: a potent stimulator of adenosine 3':5' cyclic monophosphate accumulation in gut carcinoma cell lines in culture. Proc Natl Acad Sci USA. (1978) 75:2772–5. 10.1073/pnas.75.6.2772 208077 PMC392646

[33] Maoret JJ Pospaï D Rouyer-Fessard C Couvineau A Laboisse C Voisin T. Neurotensin receptor and its mRNA are expressed in many human colon cancer cell lines but not in normal colonic epithelium: binding studies and RT-PCR experiments. Biochem Biophys Res Commun. (1994) 203:465–71. 10.1006/bbrc.1994.2205 7521165

[34] Singh P Dai B Wu H Owlia A. Role of autocrine and endocrine gastrin-like peptides in colonic carcinogenesis. Curr Opin Gastroenterol. (2000) 16:68–77. 10.1097/00001574-200001000-00013 17024020

[35] Darmoul D Gratio V Devaud H Lehy T Laburthe M. Aberrant expression and activation of the thrombin receptor protease-activated receptor-1 induces cell proliferation and motility in human colon cancer cells. Am J Pathol. (2003) 162:1503–13. 10.1016/S0002-9440(10)64283-6 12707033 PMC1851194

[36] Gratio V Walker F Lehy T Laburthe M Darmoul D. Aberrant expression of proteinase-activated receptor 4 promotes colon cancer cell proliferation through a persistent signaling that involves Src and ErbB-2 kinase. Int J Cancer (2009) 124:1517–25. 10.1002/ijc.24070 19058300

[37] Darmoul D Gratio V Devaud H Peiretti F Laburthe M. Activation of proteinase-activated receptor 1 promotes human colon cancer cell proliferation through epidermal growth factor receptor transactivation. Mol Cancer Res. (2004) 2:514–22. 15383630

[38] Yang M Zhong WW Srivastava N Slavin A Yang J Hoey T. protein-coupled lysophosphatidic acid receptor stimulate proliferation of colon cancer cells through the {beta}-catenin pathway. Proc Natl Acad Sci USA. (2005) 102:6027–32. 10.1073/pnas.0501535102 15837931 PMC1087935

[39] Chell S Kaidi A Williams AC Paraskeva C. Mediators of PGE2 synthesis and signalling downstream of COX-2 represent potential targets for the prevention/treatment of colorectal cancer. Biochim Biophys Acta (2006) 1766:104–19. 10.1016/j.bbcan.2006.05.002 16859832

[40] Darmoul D Gratio V Devaud H Laburthe M. Protease-activated receptor 2 in colon cancer: trypsin-induced MAPK phosphorylation and cell proliferation are mediated by epidermal growth factor receptor transactivation. J Biol Chem. (2004) 279:20927–34. 10.1074/jbc.M401430200 15010475

[41] Lappano R Maggiolini M. G protein-coupled receptors: novel targets for drug discovery in cancer. Nat Rev Drug Discov. (2011) 10:47–60. 10.1038/nrd3320 21193867

[42] Rouet-Benzineb P Rouyer-Fessard C Jarry A Avondo V Pouzet C Yanagisawa M. Orexins acting at native OX(1) receptor in colon cancer and neuroblastoma cells or at recombinant OX(1) receptor suppress cell growth by inducing apoptosis. J Biol Chem. (2004) 279:45875–86. 10.1074/jbc.M404136200 15310763

[43] Voisin T El Firar A Rouyer-Fessard C Gratio V Laburthe M. A hallmark of immunoreceptor, the tyrosine-based inhibitory motif ITIM, is present in the G protein-coupled receptor OX1R for orexins and drives apoptosis: a novel mechanism. FASEB J. (2008) 22:1993–2002. 10.1096/fj.07-098723 18198212

[44] Voisin T El Firar A Fasseu M Rouyer-Fessard C Descatoire V Walker F. Aberrant expression of OX1 receptors for orexins in colon cancers and liver metastases: an openable gate to apoptosis. Cancer Res. (2011) 71:3341–51. 10.1158/0008-5472.CAN-10-3473 21415167

[45] Lesuffleur T Kornowski A Luccioni C Muleris M Barbat A Beaumatin J. Adaptation to 5-fluorouracil of the heterogeneous human colon tumor cell line HT-29 results in the selection of cells committed to differentiation. Int J Cancer (1991) 49:721–30. 10.1002/ijc.2910490516 1937958

[46] Dayot S Speisky D Couvelard A Bourgoin P Gratio V Cros J. *In vitro, in vivo* and *ex vivo* demonstration of the antitumoral role of hypocretin-1/orexin-A and almorexant in pancreatic ductal adenocarcinoma. Oncotarget (2018) 9:6952–67. 10.18632/oncotarget.24084 29467942 PMC5805528

[47] Chartrel N Anouar Y Jeandel L Alexandre D Leprince J Couvineau A (2017) Methods and Pharmaceutical Compositions Using Orexins (OXA, OXB) for the Treatment of Prostate Cancers. US Patent Application 20170319661, Geneva.

[48] El Firar A Voisin T Rouyer-Fessard C Ostuni MA Couvineau A Laburthe M. Discovery of a functional immunoreceptor tyrosine-based switch motif in a 7-transmembrane-spanning receptor: role in the orexin receptor OX1R-driven apoptosis. FASEB J (2009) 23:4069–80. 10.1096/fj.09-131367 19661287

[49] Daëron M Jaeger S Du Pasquier L Vivier E. Immunoreceptor tyrosine-based inhibition motifs: a quest in the past and future. Immunol Rev. (2008) 224:11–43. 10.1111/j.1600-065X.2008.00666.x 18759918

[50] Ryan DP Hong TS Bardeesy N Pancreatic adenocarcinoma. N Engl J Med. (2014) 371:2140–41. 10.1056/NEJMra1404198 25427123

[51] Neuzillet C Tijeras-Raballand A Bourget P Cros J Couvelard A Sauvanet A. State of the art and future directions of pancreatic ductal adenocarcinoma therapy. Pharmacol Ther. (2015) 155:80–104. 10.1016/j.pharmthera.2015.08.006 26299994

[52] Rahib L Smith BD Aizenberg R Rosenzweig AB Fleshman JM Matrisian LM. Projecting cancer incidence and deaths to 2030: the unexpected burden of thyroid, liver, and pancreas cancers in the United States. Cancer Res (2014) 74:2913–21. 10.1158/0008-5472.CAN-14-0155 24840647

[53] Garcea G Neal CP Pattenden CJ Steward WP Berry DP. Molecular prognostic markers in pancreatic cancer: a systematic review. Eur J Cancer (2005) 41:2213–36. 10.1016/j.ejca.2005.04.044 16146690

[54] Shields MA Dangi-Garimella S Redig AJ Munshi HG. Biochemical role of the collagen-rich tumour microenvironment in pancreatic cancer progression Biochem J. (2012) 441:541–52. 10.1042/BJ20111240 22187935 PMC8215985

[55] Olive KP Jacobetz MA Davidson CJ Gopinathan A McIntyre D Honess D. Inhibition of hedgehog signaling enhances delivery of chemotherapy in a mouse model of pancreatic cancer. Science (2009) 324:1457–61. 10.1126/science.1171362 19460966 PMC2998180

[56] Messal N Fernandez N Dayot S Gratio V Nicole P Prochasson C Ectopic expression of OX1R in ulcerative colitis mediates anti-inflammatory effect of orexin-A. BBA Mole Basis Dis. (2018) 1864:3618–28. 10.1016/j.bbadis.2018.08.023 30251681

[57] Winrow CJ Renger JJ. Discovery and development of orexin receptor antagonists as therapeutics for insomnia. Br J Pharmacol. (2014) 171:283–93. 10.1111/bph.12261 23731216 PMC3904252

[58] Roecker AJ Cox CD Coleman PJ. Orexin receptor antagonists: new therapeutic agents for the treatment of insomnia. J Med Chem. (2016) 59:504–30. 10.1021/acs.jmedchem.5b00832 26317591

[59] Dugovic C Shelton JE Aluisio LE Fraser IC Jiang X Sutton SW. Blockade of orexin-1 receptors attenuates orexin-2 receptor antagonism-induced sleep promotion in the rat. J Pharmacol Exp Ther. (2009) 330:142–51. 10.1124/jpet.109.152009 19363060

[60] Smart D Sabido-David C Brough SJ Jewitt F Johns A Porter RA. SB-334867-A: the first selective orexin-1 receptor antagonist. Br J Pharmacol. (2001) 132:1179–82. 10.1038/sj.bjp.0703953 11250867 PMC1572677

[61] Bonaventure P Shelton J Yun S Nepomuceno D Sutton S Aluisio L. Characterization of JNJ-42847922, a selective orexin-2 receptor antagonist, as a clinical candidate for the treatment of insomnia. J Pharmacol Exp Ther. (2015) 354:471–82. 10.1124/jpet.115.225466 26177655

[62] Betschart C Hintermann S Behnke D Cotesta S Fendt M Gee CE. Identification of a novel series of orexin receptor antagonists with a distinct effect on sleep architecture for the treatment of insomnia. J Med Chem. (2013) 56:7590–607. 10.1021/jm4007627 23964859

[63] Cox CD Breslin MJ Whitman DB Schreier JD McGaughey GB Bogusky MJ. Discovery of the dual orexin receptor antagonist [(7R)-4-(5-chloro-1,3-benzoxazol-2-yl)-7-methyl-1,4-diazepan-1-yl][5-methyl-2-(2H−1,2,3-triazol-2-yl)phenyl]methanone (MK-4305) for the treatment of insomnia. J Med Chem. (2010) 53:5320–32. 10.1021/jm100541c 20565075

[64] Turku A Rinne MK Boije Af Gennäs G Xhaard H Lindholm D. Orexin receptor agonist Yan 7874 is a weak agonist of orexin/hypocretin receptors and shows orexin receptor-independent cytotoxicity. PLoS ONE (2017) 12:e0178526. 10.1371/journal.pone.0178526 28575023 PMC5456073

[65] Irukayama-Tomobe Y Ogawa Y Tominaga H Ishikawa Y Hosokawa N Ambai S. Nonpeptide orexin type-2 receptor agonist ameliorates narcolepsy-cataplexy symptoms in mouse models. Proc Natl Acad Sci USA. (2017) 114:5731–6. 10.1073/pnas.1700499114 28507129 PMC5465922

[66] Heifetz A Bodkin MJ Biggin PC. Discovery of the first selective, nonpeptidic orexin 2 receptor agonists. J Med Chem. (2015) 58:7928–30. 10.1021/acs.jmedchem.5b01394 26375584

[67] Soffin EM Gill CH Brough SJ Jerman JC Davies CH. Pharmacological characterisation of the orexin receptor subtype mediating postsynaptic excitation in the rat dorsal raphe nucleus. Neuropharmacology (2004) 46:1168–76. 10.1016/j.neuropharm.2004.02.014 15111023

[68] Smith JS Lefkowitz RJ Rajagopal S. Biased signalling: from simple switches to allosteric microprocessors. Nat Rev Drug Discov. (2018) 17:243–60. 10.1038/nrd.2017.229 29302067 PMC5936084

[69] Siegel RL Miller KD Jemal A Cancer statistics, 2016. CA Cancer J Clin. (2016) 66:7–30. 10.3322/caac.21332 26742998

[70] Patel A Fong L. Immunotherapy for prostate cancer: where do we go from here?-PART 2: checkpoint inhibitors, immunotherapy combinations, tumor microenvironment modulation, and cellular therapies. Oncology (2018) 32:e65–73. 29940064

[71] Rove KO Crawford ED. Traditional androgen ablation approaches to advanced prostate cancer: new insights. Can J Urol. (2014) 21:14–21. 24775719

[72] Watson PA Arora VK Sawyers CL. Emerging mechanisms of resistance to androgen receptor inhibitors in prostate cancer. Nat Rev Cancer (2015) 15:701–11. 10.1038/nrc4016 26563462 PMC4771416

[73] Wang W Chen ZX Guo DY Tao YX. Regulation of prostate cancer by hormone-responsive G protein-coupled receptors. Pharmacol Ther. (2018) [Epub ahead of print]. 10.1016/j.pharmthera.2018.06.005 29909235

[74] Maly IV Hofmann WA. Calcium and nuclear signaling in prostate cancer. Int J Mol Sci. (2018) 19:E1237. 10.3390/ijms19041237 29671777 PMC5979488

[75] Li S Huang S Peng SB. Overexpression of G protein-coupled receptors in cancer cells: involvement in tumor progression. Int J Oncol. (2005) 27:1329–39. 10.3892/ijo.27.5.1329 16211229

[76] Sumitomo M Shen R Walburg M Dai J Geng Y Navarro D. Neutral endopeptidase inhibits prostate cancer cell migration by blocking focal adhesion kinase signaling. J Clin Invest. (2000) 106:1399–407. 10.1172/JCI10536 11104793 PMC381465

[77] Collado B Sanchez MG Diaz-Laviada I Prieto JC Carmena MJ. Vasoactive intestinal peptide (VIP) induces c-fos expression in LNCaP prostate cancer cells through a mechanism that involves Ca^2+^ signalling. Implications in angiogenesis and neuroendocrine differentiation. Biochim Biophys Acta (2005) 1744:224–33. 10.1016/j.bbamcr.2005.04.009 15921770

[78] Farini D Puglianiello A Mammi C Siracusa G Moretti C. Dual effect of pituitary adenylate cyclase activating polypeptide on prostate tumor LNCaP cells: short- and long-term exposure affect proliferation and neuroendocrine differentiation. Endocrinology (2003) 144:1631–43. 10.1210/en.2002-221009 12639948

[79] Juarranz MG Bolanos O Gutierrez-Canas I Lerner EA Robberecht P Carmena MJ. Neuroendocrine differentiation of the LNCaP prostate cancer cell line maintains the expression and function of VIP and PACAP receptors. Cell Signal. (2001) 13:887–94. 10.1016/S0898-6568(01)00199-1 11728828

[80] Abasolo I Montuenga LM Calvo A. Adrenomedullin prevents apoptosis in prostate cancer cells. Regul Pept. (2006) 133:115–22. 10.1016/j.regpep.2005.09.026 16297990

[81] Zhong M Boseman ML Millena AC Khan SA. Oxytocin induces the migration of prostate cancer cells: involvement of the Gi-coupled signaling pathway. Mol Cancer Res. (2010) 8:1164–72. 10.1158/1541-7786.MCR-09-0329 20663860 PMC2923666

[82] Bhola NE Grandis JE. Crosstalk between G-protein-coupled receptors and Epidermal growth factor receptor in cancer. Front. Biosci. (2008) 13:1857–65. 10.2741/2805 17981673

[83] Alexandre D Hautot C Mehio M Jeandel L Courel M Voisin T. The orexin type 1 receptor is overexpressed in advanced prostate cancer with a neuroendocrine differentiation, and mediates apoptosis. Eur J Cancer (2014) 50:2126–33. 10.1016/j.ejca.2014.05.008 24910418

[84] Malendowicz W Szyszka M Ziolkowska A Rucinski M Kwias Z. Elevated expression of orexin receptor 2 (HCRTR2) in benign prostatic hyperplasia is accompanied by lowered serum orexin A concentrations. Int J Mol Med. (2011) 27:377–83. 10.3892/ijmm.2010.590 21186399

[85] Valiante S Liguori G Tafuri S Pavone LM Campese R Monaco R. Expression and potential role of the peptide orexin-A in prostate cancer. Biochem Biophys Res Commun. (2015) 464:1290–6. 10.1016/j.bbrc.2015.07.124 26220343

[86] Tsunematsu T Yamanaka A. The role of orexin/hypocretin in the central nervous system and peripheral tissues. Vitam Horm. (2012) 89:19–33. 10.1016/B978-0-12-394623-2.00002-0 22640606

[87] Graybill NL Weissig V. A review of orexin's unprecedented potential as a novel, highly-specific treatment for various localized and metastatic cancers. SAGE Open Med. (2017) 5:1–9. 10.1177/2050312117735774 29147564 PMC5673000

[88] Wenzel J Grabinski N Knopp CA Dendorfer A Ramanjaneya M Randeva HS. Hypocretin/orexin increases the expression of steroidogenic enzymes in human adrenocortical NCI H295R cells. Am J Physiol Regul Integr Comp Physiol. (2009) 297:R1601–9. 10.1152/ajpregu.91034.2008 19793950

[89] Spinazzi R Rucinski M Neri G Malendowicz LK Nussdorfer GG. Preproorexin and orexin receptors are expressed in cortisol-secreting adrenocortical adenomas, and orexins stimulate *in vitro* cortisol secretion and growth of tumor cells. J Clin Endocrinol Metab. (2005) 90:3544–9. 10.1210/jc.2004-2385 15797953

[90] Liu Y Zhao Y Guo L. Effects of orexin A on glucose metabolism in human hepatocellular carcinoma *in vitro* via PI3K/Akt/mTOR-dependent and -independent mechanism. Mol Cell Endocrinol. (2016) 420:208–16. 10.1016/j.mce.2015.11.002 26549689

[91] Liu Y Zhao Y Ju S Guo L. Orexin A upregulates the protein expression of OX1R and enhances the proliferation of SGC-7901 gastric cancer cells through the ERK signaling pathway. Int J Mol Med. (2015) 35:539–45. 10.3892/ijmm.2014.2038 25515760

[92] Wen J Zhao Y Shen Y Guo L. Effect of orexin A on apoptosis in BGC-823 gastric cancer cells via OX1R through the AKT signaling pathway. Mol Med Rep. (2015) 11:3439–44. 10.3892/mmr.2015.3190 25586545

[93] Bai B Chen X Zhang R Wang X Jiang Y Li D. Dual-agonist occupancy of orexin receptor 1 and cholecystokinin A receptor heterodimers decreases G-protein-dependent signaling and migration in the human colon cancer cell line HT-29. Biochim Biophys Acta (2017) 1864:1153–64. 10.1016/j.bbamcr.2017.03.003 28288880

[94] Mazzocchi G Malendowicz LK Aragona F Rebuffat P Gottardo L Nussdorfer GG. Human pheochromocytomas express orexin receptor type 2 gene and display an *in vitro* secretory response to orexins A and B. J Clin Endocrinol Metab. (2001) 86:4818–21. 10.1210/jcem.86.10.7929 11600547

[95] Nanmoku T Isobe K Sakurai T Yamanaka A Takekoshi K Kawakami Y. Orexins suppress catecholamine synthesis and secretion in cultured PC12 cells. Biochem Biophys Res Commun. (2000) 274:310–5. 10.1006/bbrc.2000.3137 10913336

[96] Dehan P Canon C Trooskens G Rehli M Munaut C Van Criekinge W. Expression of type 2 orexin receptor in human endometrium and its epigenetic silencing in endometrial cancer. J Clin Endocrinol Metab. (2013) 98:1549–57. 10.1210/jc.2012-3263 23482607

[97] Kantono M Guo B. Inflammasomes and cancer: the dynamic role of the inflammasome in tumor development. Front Immunol. (2017) 8:1132. 10.3389/fimmu.2017.01132 28955343 PMC5600922

[98] Duffy C M Yuan C Wisdorf L E Billington CJ Kotz CM Nixon JP. Role of orexin A signaling in dietary palmitic acid-activated microglial cells. Neurosci Lett. (2015) 606:140–4. 10.1016/j.neulet.2015.08.033 26306651 PMC4811357

[99] Xiong X White RE Xu L Yang L Sun X Zou B. Mitigation of murine focal cerebral ischemia by the hypocretin/orexin system is associated with reduced inflammation. Stroke (2013) 44:764–70. 10.1161/STROKEAHA.112.681700 23349191 PMC3638929

[100] Ogawa Y Irukayama-Tomobe Y Murakoshi N Kiyama M Ishikawa Y Hosokawa N. Peripherally administered orexin improves survival of mice with endotoxin shock. Elife (2016) 5:e21055. 10.7554/eLife.21055 28035899 PMC5245965

[101] Chieffi S Carotenuto M Monda V Valenzano A Villano I Precenzano F. Orexin system: the key for a healthy life. Front Physiol. (2017) 8:357. 10.3389/fphys.2017.00357 28620314 PMC5450021

[102] Prochasson C Bergere M Messal N Dayot S Rebours V Voisin T The hypothalamic neuropeptide, orexin, prevents chronic pancreatitis in cerulein mice model. Gastroenterology (2016) 150:S917 10.1016/S0016-5085(16)33110-9

[103] Martínez-Orozco FJ Vicario JL Villalibre-Valderrey I De Andrés C Fernández-Arquero M Peraita-Adrados R. Narcolepsy with cataplexy and comorbid immunopathological diseases. J Sleep Res. (2014) 23:414–9. 10.1111/jsr.12143 24645699

